# Promoting the use of self-management in novice chiropractors treating individuals with spine pain: the design of a theory-based knowledge translation intervention

**DOI:** 10.1186/s12891-018-2241-1

**Published:** 2018-09-11

**Authors:** Owis Eilayyan, Aliki Thomas, Marie-Christine Hallé, Sara Ahmed, Anthony C. Tibbles, Craig Jacobs, Silvano Mior, Connie Davis, Roni Evans, Michael J. Schneider, Fadi Alzoubi, Jan Barnsley, Cynthia R. Long, Andre Bussières

**Affiliations:** 1 School of Physical and Occupational Therapy, McGill University, 3654 Prom Sir-William-Osler, Montréal, QC H3G 1Y5 Canada; 2 Center for Interdisciplinary Research in Rehabilitation of Greater Montreal (CRIR), Montréal, Canada; 3 Canadian Memorial Chiropractic College, North York, Canada; 4 University of British Columbia, Vancouver, Canada; 5 Centre for Collaboration, Motivation and Innovation, Vancouver, Canada; 6 University of Minnesota, Minneapolis, USA; 7 University of Pittsburgh, Pittsburgh, USA; 8 University of Toronto, Toronto, Canada; 9 Palmer College Davenport, Davenport, USA

**Keywords:** Spine pain, Self-management, Theory-based intervention, Knowledge translation, Theoretical domain framework, Chiropractic, Brief action planning

## Abstract

**Background:**

Clinical practice guidelines generally recommend clinicians use self-management support (SMS) when managing patients with spine pain. However, even within the educational setting, the implementation of SMS remains suboptimal. The objectives of this study were to 1) estimate the organizational readiness for change toward using SMS at the Canadian Memorial Chiropractic College (CMCC), Toronto, Ontario from the perspective of directors and deans, 2) estimate the attitudes and self-reported behaviours towards using evidence-based practice (EBP), and beliefs about pain management among supervisory clinicians and chiropractic interns, 3) identify potential barriers and enablers to using SMS, and 4) design a theory-based tailored Knowledge Translation (KT) intervention to increase the use of SMS.

**Methods:**

Mixed method design. We administered three self-administered questionnaires to assess clinicians’ and interns’ attitudes and behaviours toward EBP, beliefs about pain management, and practice style. In addition, we conducted 3 focus groups with clinicians and interns based on the Theoretical Domain Framework (TDF) to explore their beliefs about using SMS for patients with spine pain. Data were analysed using deductive thematic analysis by 2 independent assessors. A panel of 7 experts mapped behaviour change techniques to key barriers identified informing the design of a KT intervention.

**Results:**

Participants showed high level of EBP knowledge, positive attitude of EBP, and moderate frequency of EBP use. A number of barrier factors were identified from clinicians (*N* = 6) and interns (*N* = 16) corresponding to 7 TDF domains: *Knowledge; Skills; Environmental context and resources; Emotion; Beliefs about Capabilities; Memory, attention & decision making;* and *Social Influence*. To address these barriers, the expert panel proposed a multifaceted KT intervention composed of a webinar and online educational module on a SMS guided by the Brief Action Planning, clinical vignettes, training workshop, and opinion leader support.

**Conclusion:**

SMS strategies can help maximizing the health care services for patients with spine pain. This may in turn optimize patients’ health. The proposed theory-based KT intervention may facilitate the implementation of SMS among clinicians and interns.

**Electronic supplementary material:**

The online version of this article (10.1186/s12891-018-2241-1) contains supplementary material, which is available to authorized users.

## Background

Spine pain is very common and is a leading cause of disability worldwide [1–5]. Between 50 and 80% of adults suffer from spine pain during their lives [6, 7], which is associated with a high individual (physical, psychological, emotional) and societal burden [6, 8–16]. In Canada, the estimated direct cost of spine pain ranges from $6 to $12 billion annually [17].

Many people with spine pain consult chiropractors for pain relief [18–20]. Clinical practice guidelines (CPGs) generally recommend offering self-management support (SMS) strategies to individuals with spine pain [21–28] as these help reduce the associated individual and societal burden [29]. SMS strategies are designed to facilitate adoption of healthy lifestyle in people with a range of health issues including spine pain and related co-morbidities (e.g. heart disease, type 2 diabetes, depression) [30–39]. In patients with spine pain, SMS can help decrease levels of pain, disability, and psychological distress [40, 41]. However, the routine adoption of evidence-based practices (EBPs) including the use of CPGs remains suboptimal among care providers including chiropractors [42–45]. Barriers to implementing EBPs among chiropractors include: lack of time, lack of generalizability of guidelines, lack of compensation, time since graduation greater than 10 years, insufficient skills or confidence in using findings from the literature, predefined beliefs and a more narrowed scope of practice [43].

SMS interventions empower the patient to be efficiently involved in their own care by involving them in the decision-making process [46, 47]. SMS strategies also necessitate a close collaboration between clinicians and patients [47–49]. However, a number of barriers to implementing SMS among clinicians have been documented, including: the lack of sufficient knowledge and skills to empower patients or to provide them with useful information, lack of time, and unfavourable patient views about this approach. Inadequate communication between clinicians and patients may also limit the use of SMS [50–53]. In addition, organizational barriers could restrict the use of SMS in clinical settings, such as patient overload, short treatment session, and long waiting lists [53]. Together, these barriers can contribute to reducing the effectiveness of SMS. Given the documented barriers to adoption of EBPs - and SMS in particular - changing clinicians’ behaviour is challenging [43, 54].

Knowledge Translation (KT) is an approach used to facilitate’ behavioural change in practitioners [55]. It can be used to promote the early use of EBP and CPGs during professional training, which may be more effective than changing existing professional practice to support the long-term use of best evidence [56, 57]. EBP requires the integration of research evidence, clinical expertise and patients’ preferences into clinical decision-making [58]. Systematic reviews suggest that, while classroom-based teaching primarily improves EBP knowledge, clinically integrated teaching of EBP may be the most effective approach for improving the knowledge, attitudes, skills and behaviours associated with the use of EBP. Thus, academic programs must first lay down the foundations of EBP over the course of professional training, and then move students along a trajectory of progressive development of EBP competencies [56, 59]. Clinically integrated teaching of EBP delivered in the clinical setting can support deeper reflection on practice through actual patient management [60, 61].

Thus, providing chiropractic interns with the opportunity to routinely use CPGs to inform their clinical decisions should increase the likelihood of uptake and sustained use of EBP in their future practices. These interns will be more likely to become lifelong learners and reflective practitioners who will be equipped to overcome barriers to the use of CPGs - including SMS – and contribute to reducing research-practice gaps [43].

In Canada, the majority of practising chiropractors (58%) are trained at the Canadian Memorial Chiropractic College (CMCC) [62]. While CMCC revised its curriculum to promote the sustainable use of EBP among graduates, structured SMS that allows for patient-centred goals such as the Brief Action Planning (BAP) [63] has not yet been integrated into the curriculum [64]. Consequently, supervisory clinicians and interns do not systematically use SMS with patients across the CMCC outpatient teaching clinics [64].

The objectives of this study were to 1) estimate the organizational readiness for change toward using SMS at the Canadian Memorial Chiropractic College (CMCC), Toronto, Ontario from the perspective of Directors and Deans, 2) estimate the attitudes towards and self-reported use of evidence-based practice (EBP) behaviours, as well as beliefs about pain management among supervisory clinicians and chiropractic interns, 3) identify potential barriers and enablers to using SMS, and 4) design a theory-based tailored Knowledge Translation (KT) intervention to increase the use of SMS.

### Conceptual framework

The Theoretical Domain Framework (TDF) has been used across several health disciplines, settings, and conditions to assess barriers to change and guide the development of theory-based interventions [65–69]. The TDF covers the main factors that influence behaviour change in clinical practice: *Knowledge, Skills, Social/Professional Role and Identity, Beliefs about Capabilities, Optimism, Beliefs about Consequences, Reinforcement, Intentions, Goals, Memory/Attention and Decision Processes, Environmental Context and Resources, Social Influences, Emotion,* and *Behavioural Regulation* [70].

## Materials

### Study design

Mixed-methods sequential transformative design comprising both quantitative and qualitative analyses. Ethical approval was obtained from the Research Ethics Board of McGill University (McGill IRB: A08-E54-16B), and written informed consent was obtained from all participants.

### Setting

Five outpatient-teaching clinics of the Canadian Memorial Chiropractic College (CMCC), a major teaching institution in Ontario were approached to participate in the study.

The development of a KT intervention aiming to promote the use of SMS was guided by a systematic approach proposed by French et al. (2012) [69]. The approach includes 4 questions:

1. Who needs to do what, differently? (i.e. identify the evidence-practice gap). For this question, the literature suggests that the use of SMS among clinicians is suboptimal [50–53].
2. Using a theoretical framework (i.e. TDF [70]), which barriers and enablers need to be addressed? and
3. Which intervention components (behaviour change techniques and mode(s) of delivery) could overcome the modifiable barriers and enhance the enablers?

The latter 2 questions were addressed in two separate phases: Phase 1A aimed to 1) explore CMCC organizational readiness to use of EBP and SMS (Quantitative), 2) explore clinicians’ and interns’ behaviours and attitudes towards the use of EBP and their beliefs about pain management (Quantitative). Phase 1B aimed to identify barriers and enablers to the use of SMS among a subgroup of clinicians and interns who were representative of CMCC clinicians and interns in terms of age, gender, and years of experience (Qualitative). Results from phase 1 were integrated and used to inform phase 2, where we mapped key barriers to using SMS. Ultimately, the findings served to design KT intervention components to address these barriers.

4) How can behaviour change be measured and understood? This question is beyond the scope of this paper.

#### Phase 1A targeting objectives 1 and 2: Clinicians’ and interns’ behaviours and attitudes toward EBP use, and the organizational readiness for change in healthcare settings (Quantitative Data)

##### Participants

Chiropractic interns working within 20 Patient Management Teams (PMTs) and their 20 supervisory clinicians were invited to participate in this phase. Chiropractic interns had to be in their final year at CMCC and working in one of these 20 PMTs. Directors and deans at CMCC (decision makers) were also invited to participate in the study.

##### Data collection

Study instruments

The decision makers at CMCC completed the Organizational Readiness for Implementing Change (ORIC) questionnaire which assesses organizational readiness for change in healthcare settings [71]. Clinicians and interns completed 3 self-administered questionnaires: 1) The Knowledge, Attitude, and Behaviour Questionnaire (KABQ) that assesses knowledge, attitudes, and behaviour toward EBP [72], 2) the Pain Attitudes and Beliefs Scale (PABS) which assesses the strength of 2 treatment orientations of health care practitioners: biomedical and behavioural orientations [73], and 3) the practice style questionnaire to classify clinicians and interns based on their practice [74].

##### Organizational readiness for implementing change (ORIC)

The ORIC is comprised of 12 questions forming 2 domains: change commitment and change efficacy [71]. Each question is rated on 5-point Likert scale (Strongly Disagree – Strongly Agree), scores range 12–60, with higher scores indicating high readiness for change among organization members [71]. The ORIC has good psychometric properties [71].

##### Knowledge, attitude, and behaviour questionnaire (KABQ)

The KABQ is a 33–item validated questionnaire comprised of 4 EBP domains: knowledge, attitudes, behaviours and outcomes/decisions [72]. The ‘knowledge’ domain includes 8 items each rated on a 7-point Likert scale, with higher scores indicating a higher level of EBP knowledge. The ‘attitudes towards EBP’ domain contains 14 items rated on a 7-point Likert scale, with higher scores indicating more positive attitudes toward EBP. The “Behaviour towards EBP” domain includes 8 items rated on a 5-point Likert scale, with higher scores indicating a higher frequency of using EBP. Lastly, the “outcomes/decisions” domain includes 3 items rated on a 6-point Likert scale, with lower scores indicating less favourable patient outcomes and poorer clinical evidence-based decision making [72]. This questionnaire has demonstrated good psychometric properties [72].

##### Pain attitudes and beliefs scale (PABS)

The PABS questionnaire assesses the strength of 2 treatment orientations of health care practitioners: biomedical and behavioural orientations [73]. The amended version of the PABS is comprised of 19 items (10 biomedical items and 9 behavioural items) [75]. Each question is rated on a 6-point scale “(‘Totally disagree’ = 1 to ‘Totally agree’ = 6)”, where higher scores on a subscale indicate a stronger treatment orientation [75]. The PABS has acceptable psychometric properties [73, 76].

##### Practice style questionnaire

The practice style questionnaire is used to classify clinicians into 4 categories based on their style of practice: Seekers, Receptives, Traditionalists, and Pragmatists [74]. The questionnaire includes 17 statements about clinicians’ practice rated on 5-point Likert scale (Strongly Agree – Strongly Disagree).

##### Procedure

A member of the research team and Director of Clinical Education and Patient Care at CMCC (C.J.) personally introduced the study to the CMCC decision makers (*N* = 20) and at a faculty meeting. Decision makers who agreed to participate in the study completed the ORIC tool online.

We first pilot tested the KABQ, PABS, and practice style questionnaires with one volunteering PMT composed of a supervisory clinician and seven interns. Team member (C.J.) sent these PMT participants an email with a link to the online survey along with a feedback form. Respondents were invited to indicate the length of time needed to complete the questionnaire and any questions or comments they had regarding the clarity of the questionnaires. Feedback received allowed the research team to correct typographical errors and develop an appendix providing additional clarifications for a few questions for which the wording or the meaning appeared to be confusing. C.J. then sent an email to all supervisory clinicians (*N* = 20) and interns (*N* = 173) of the remaining PMTs informing them about the study (e.g., goal, timeline and procedures) and inviting them to dedicate half an hour of their administrative time to complete the questionnaires in the upcoming week. To avoid coercion, clinicians were invited to complete the same questionnaires at the same time as their interns, but in a different room. All supervisory clinicians and interns received the link to the online surveys and the appendix providing additional clarifications about the surveys via an email sent by C.J. An online consent form preceded the surveys.

##### Sample size and data analysis

Descriptive analysis was conducted for the 4 administered questionnaires using SAS 9.4 [77]. The scores were calculated for each subscale of the KABQ, PABS, and ORIC. For the practice style questionnaire, the frequency of each category was calculated. The associations between demographic variables and the sub-scores/total score of each questionnaire were assessed using simple and multiple linear regression models. The socio-demographic variables included age, gender, education, grade point average (GPA), and clinical experience. *Β*-coefficients were used to assess the association between KABQ and PABS with other factors. All studied factors were considered as categorical variables with the exception of age, which was a continuous variable.

Sample size in multiple regression depends upon the number of studied variables following the rule of thumb of (*N* ≥ 50 + 8 m), where *m* refers to number of studied (predictors) variables [78]. As this study included 5 predictors, a sample size of 106 subjects was needed to run a multiple linear regression with an alpha of 0.05 and 80% power (as a function of medium effect size) [78, 79].

#### Phase 1B: Barriers and enablers to the use SMS (qualitative)

We conducted three 90-min focus groups with a subset of supervisory chiropractors and interns to identify the key barriers and enablers to the use SMS.

##### Focus group guide

The interview topic guide was developed based on the TDF framework [70] and further informed by our previous work [80–84]. The topic guide included 27 open-ended questions which covered all 14 TDF domains, with on average 2–3 questions per domain. Probing questions were used for further clarification if needed (See Additional file 1). Each focus group took approximately 90 min.

##### Procedure

A member of the research team (C.J.) sent an invitation email to all clinicians and interns at CMCC to participate in a focus group. The email included a link to an online form requesting potential participants’ authorization to be contacted by the research team and asking them to provide their name, contact information and a few socio-demographic information. Three focus groups were conducted: one with 6 clinicians, and two with 8 interns each. All focus groups took place in person at CMCC. A research assistant, experienced in conducting qualitative interviews based on the TDF, facilitated the focus groups. All participants completed and signed a consent form prior to the focus groups. Each focus group took approximately 90 min, was audio recorded, anonymized and transcribed verbatim.

##### Data analysis

The analysis in this study followed the same analysis used by the research team previously [68, 82]. The focus group data were coded deductively by 2 independent reviewers (HO & OE). Disagreements were resolved by 2 other team members who have previous experience with using the TDF (AB and FZ). Each transcript was divided into different statements that were coded into relevant TDF domains. Statements were then linked with specific beliefs. A specific belief is defined as “a core statement that captures a common theme from multiple response statements and provides detail about the role of a given domain in influencing practice behaviour” [68, 80]. The specific beliefs were classified into one of 3 categories based on the likelihood that they would 1) increase (facilitator), 2) decrease (barrier), or 3) have no influence on the use of SMS. Similar specific beliefs within each TDF domain were identified and grouped into overarching themes. Three criteria were used concurrently to identify the key barriers: frequency of belief, importance of the belief, and contrasting beliefs.

##### Sample size

The sample size needed for deriving thematic saturation from focus groups cannot be determined in advance. The literature suggests having 2–3 focus groups, a size of 8 participants each to discover most of the themes about the studied area [85]. There was no a priori plan to assess the saturation of focus group data. However, both clinicians and interns indicated almost identical barriers to using SMS.

##### Phase 2: Intervention design

The aim of phase 2 was to review the key barriers identified in phase 1 in order to inform the design of a KT intervention to address these barriers.

##### Participants

Seven research team members with experience using Behaviour Change Techniques (BCTs) and the TDF attended a half-day meeting to consider and propose possible KT intervention components. The team included 3 KT researchers, a researcher in medical education, 2 CMCC faculty members, and 1 patient representative.

##### Procedure

All possible KT intervention components were first selected by a subgroup of 3 team members (AB, AT, OE) after mapping key TDF barriers onto corresponding BCTs (as per Michie et al. [86, 87]). Other team members received the results of this mapping exercise for consideration prior to the group meeting. Findings were reviewed by the team members, and they were asked to brainstorm other possible KT intervention components. Consensus on the selection of KT intervention components and modes of delivery was reached based on the evidence of their effectiveness and the feasibility of implementation.

The selected KT interventions in this study that aimed to promote the use of SMS were guided by Brief Action Planning (BAP) framework [63]. The literature supported the use of the BAP framework to enable the implementation of SMS [63, 88]. The framework was developed based on motivational interviewing, and it was considered an excellent SMS program for busy clinics [63].

## Results

### Phase 1A— ORIC, KABQ, BAPS and practice style

The data set included 12 decision makers, 14 clinicians, 115 chiropractic interns, with a mean age of 57 ± 6.3 years, 46 ± 12 years and 27 ± 2.4 years, respectively. Twenty-five percent of decision makers, 14% of clinicians, and 46% of interns and were females. The raw data are presented in Additional files 2 & 3.

**Fig. 1:**
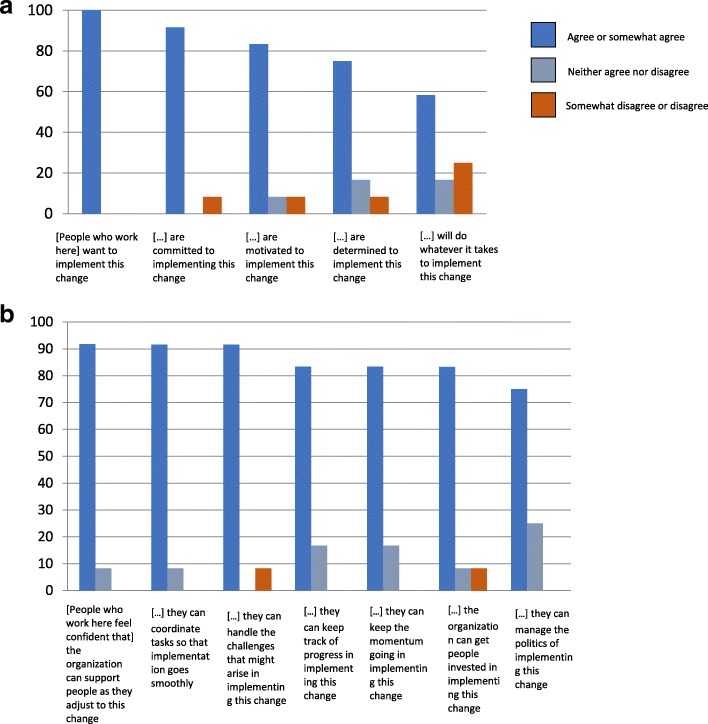
Response frequency (%) on ORIC. **a** Response frequency (%) on the “Change commitment subscale” of the ORIC, **b** Response frequency (%) on the “Change efficacy (confidence) subscale” of the ORIC. Agree or somewhat agree, Neither agree nor disagree, Somewhat disagree or disagree

Results from the ORIC showed that decision makers perceived that members of the CMCC were highly committed (mean = 20.6 ± 3.5) to, and confident about (mean = 29.3 ± 4) implementing SMS for patients with spine pain in CMCC outpatient teaching clinics, Fig. 1 A&B.

**Fig. 2:**
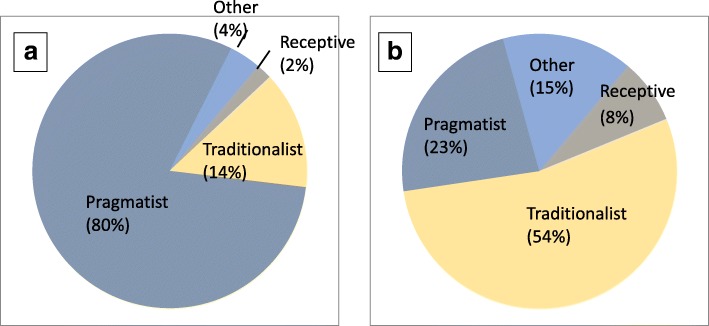
Interns’ and clinicians’ practice style trait. **a** Interns’ practice style trait, **b** Clinicians’ practice style trait

Results from the KABQ revealed that both clinicians and interns had high levels of knowledge about EBP (Clinician mean = 29.1 ± 3.7, Intern mean = 28.5 ± 4), positive attitudes towards the use of EBP (Clinician mean = 50.1 ± 6.2, Intern mean = 54.4 ± 5.4) and moderate frequency of using EBP (Clinician mean = 12.9 ± 3.3, Intern mean = 12.8 ± 2.8). The participants reported having favourable patient outcomes and good clinical evidence-based decision-making (Clinician mean = 13.3 ± 2.9, Intern mean = 12.1 ± 2.2). While interns had a significantly stronger behavioural than biomedical treatment orientation, clinicians did not show a significant difference in treatment orientation (Table 1). Lastly, 54% (7/13) of clinicians have a traditional practice style (their intervention decisions are guided by their clinical experience [89]), while 81% (87/108) of interns have a pragmatic practice style (their practice primarily depends on the workload [89]). Neither clinicians nor interns were classified as seekers (their intervention decisions are guided by evidence [89]), Fig. 2.

**Table 1:** Behavioural and biomedical treatment orientation among supervisory clinicians and interns

Group	Behavioural Treatment Orientation	Biomedical Treatment Orientation	*p*-value*
Clinicians (*N* = 13)	34.69 (5.7)	29.31 (7.4)	0.12
Interns (*N* = 108)	34.96 (4.3)	32.6 (5.9)	0.001

*Dependent *t* test

The multiple regression models showed that none of the demographic factors appeared to influence the interns’ self-reported use of EBP. However, the model revealed that men had significantly higher knowledge of EBP than women (*β* = 1.74, *p* = 0.043) and interns who had a previous university degree had more negative attitudes toward EBP (*β* = 4.3, *p* = 0.035). Regression analyses were not conducted on the clinician and decision-maker data due to the small sample sizes.

### Phase 1B—Focus groups

We conducted one focus group with 6 supervisory clinicians and 2 focus groups with 8 interns each. Clinicians’ and interns’ average age was 40.8 ± 6 years and 27 ± 2.8 years, respectively. Almost 33% (2/6) and 44% (7/16) of the clinicians and interns were females, respectively. Clinicians who participated in the focus group had an average of 12.7 ± 4.4 years of clinical experience.

### Key themes identified within relevant domains

We identified 720 statements from clinicians representing 38 specific beliefs and 18 themes. For interns, 509 statements were found and represented 56 specific beliefs and 22 themes (Additional files 4, 5, 6 and 7). Four key TDF domains were considered to have a greater likelihood to influence the targeted behaviour among both clinicians and interns: 1) Knowledge; 2) Skills; 3) Environmental context and resources; and 4) Emotion. In addition, another 3 key TDF domains were considered to have a greater influence on the targeted behaviour among only interns: 1) Beliefs about Capabilities; 2) Memory, attention and decision making; and 3) Social Influence.

### Key TDF domains (phase 1)

#### Shared domains by supervisory clinicians and interns

Four key TDF domains were shared by both clinicians and interns: 1) Knowledge; 2) Skills; 3) Environmental context and resources; and 4) Emotions.

### Knowledge

#### Clinicians

Sixteen statements were mapped to the knowledge domain. Three specific beliefs corresponded to the statements forming 2 themes: awareness of SMS and knowledge of SMS. Almost all clinicians stated that they did not attend a specific course on SMS, and that they had acquired a little knowledge of SMS from different courses. In addition, they said that there was a lack of a comprehensive SMS course. Most participants indicated that they were aware of SMS guidelines and evidence.

There were conflicting opinions between clinicians regarding interns’ knowledge of SMS: 3 clinicians considered that interns to lack knowledge of SMS, while 2 clinicians considered interns to have adequate knowledge of SMS.

#### Interns

Fifty-four statements were associated with the knowledge domain. Three specific beliefs corresponded to the statements forming 2 themes: awareness of SMS and knowledge of SMS. Most interns indicated that they were aware of SMS guidelines and evidence, and had enough knowledge of SMS. Few interns stated that formal SMS courses were needed.

### Skills

#### Clinicians

Twelve statements referred to the skills domain. Two specific beliefs corresponded to the statements representing one theme: skills needed to use SMS. Most clinicians stated that they needed to gain the skills required to use SMS, especially communication skills. Also, the clinicians indicated that interns had the skills needed to use SMS, as they had already attended SMS lectures.

#### Interns

Forty-one statements pertained to the skills domain. These statements formed 3 specific beliefs and one theme: skills needed to use SMS. Almost half of interns stated that they lacked the skills to use SMS efficiently, and indicated that they were not trained on SMS. Furthermore, the interns referred to the need for training courses to gain skills required to use SMS. Few interns mentioned that they lacked the skills to support behavioral change.

### Environmental context and resources

#### Clinicians

Thirty-one clinician statements were mapped on to the environmental context and resources domain. These statements represented 4 specific beliefs and formed 3 themes: 1) lack of time; 2) clinic’s characteristics; and 3) patients’ characteristics. Most of the clinicians stated that lack of time was a barrier to the use of SMS. Participants reported that the clinic’s characteristics (e.g. having rehabilitation equipment and sufficient space**,** collaborative clinicians**,** and having interns on placement) could facilitate the use of SMS among clinicians. Furthermore, clinicians indicated that patient’s characteristics could restrict the use of SMS, including patient’s lack of compliance, resources, or time; patient’s priorities; psychological overlay; not accepting the condition; not trusting the clinicians; and/or language and cultural barriers.

#### Interns

Seventy statements linked to the environmental context and resources. The statements represented 8 specific beliefs and formed 6 themes: 1) lack of time; 2) clinic’s characteristics; 3) patients’ characteristics; 4) financial issues; 5) lack of guidelines; and 6) course training. Almost half of interns stated that lack of time was a barrier to the use of SMS. Interns who participated in the focus groups listed the clinic’s characteristics that could facilitate the use of SMS: collaborative clinicians, having kinesiology students to refer patients to, and smaller caseload. On the other hand, interns indicated that certain clinic characteristics could restrict the use of SMS: lack of space and equipment, staff shortage, clinician characteristics (unaware of guidelines), lack of communication with peers, and not having enough exposure to different patient conditions. Furthermore, the interns stated that certain patient characteristics could restrict the use of SMS, including: fear avoidance behaviour, lack of patient adherence to SMS, lack of patient motivation to use SMS, and/or patient preference for passive care.

In addition, interns reported 2 additional major barriers: financial considerations and internship requirements. The interns believed that focusing on SMS and active care may result in losing patients who preferred a passive care approach. Interns were also concerned that using SMS would increase the duration of their treatment sessions, thereby causing them to see fewer patients. Regarding internship program requirements, the interns stated that the use of SMS was not a program requirement.

### Emotion

#### Clinicians

Eleven statements were associated with the emotion domain. These statements corresponded to 3 specific beliefs and formed one theme: anxiety about the use of SMS. Although almost all clinicians felt anxious when using SMS with patients who had psychological overlay, almost half of participants felt excited about using SMS. One clinician felt terrified of having self-management guidelines; he thought that this might discourage students from using their clinical judgement.

#### Interns

Thirty-three statements mapped to the emotion domain. The statements corresponded to 5 specific beliefs and formed one theme: feelings toward the use of SMS. Some interns felt concerned and frustrated when patients did not adhere to SMS or if they had psychological overlay. On the other hand, some interns felt exited and optimistic about the use of SMS. Furthermore, some interns stated that they felt disappointed because of certain clinicians’ behaviours, including: prioritizing one treatment over another, non-awareness of the guidelines, and not using SMS.

#### Key domains identified only for interns

Three additional TDF domains were identified among interns: 1) *Beliefs about Capabilities; 2) Memory, attention and decision making; and 3) Social Influence*.

### Beliefs about capabilities

The interns provided 52 statements that were associated with the beliefs about capabilities domain, representing 6 specific beliefs and 2 themes: acceptance and capabilities. Almost all interns stated that they were confident in managing spine pain using SMS, and they had the ability to use SMS. However, most interns indicated that the delivery of SMS was not easy, and the factors that could increase their level of confidence included observing patients benefits from SMS, having experience with SMS, and asking clinicians and colleagues.

### Memory, attention & decision making

Twenty-three statements were mapped to the domain of memory, attention & decision-making. The statements represented 5 specific beliefs and formed one theme: decision making on use of SMS. Most of the interns stated that their decisions on SMS varied according to patients’ needs. However, some interns mentioned that they did not follow a guideline to guide decisions on the use of SMS; one intern used intuition to decide whether or not to use SMS. Interestingly, one intern decided to not use SMS in order to keep patients coming to the clinic, as the patients preferred passive treatments. Lastly, few interns decided to refer patients with psychological overlay to other healthcare providers.

### Social influence

Thirty-four statements were related to the social influence domain. These statements corresponded to 4 specific beliefs and formed one theme: influence of others. Almost half of the interns stated that the clinicians’ perception of SMS restricted their use of SMS, while the other half mentioned that clinicians’ views facilitated their use of SMS. About half of participants mentioned that they consulted either supervisory clinicians or colleagues on the use of SMS. In addition, interns indicated that patients who preferred passive care could influence their decision to use SMS.

### Phase 2 — Final selection of knowledge translation intervention components

Additional file 8 presents the BCTs mapped onto key barriers identified. The research team members considered intervention components to facilitate the use of SMS among clinicians and interns for patients with spine pain, based on current evidence and feasibility of implementation at CMCC clinics. The proposed intervention includes 6 components: 1) supportive handouts summarizing how to use the SMS guided by the BAP; 2) webinar describing the benefits of using SMS and the BAP in particular; 3) an online educational module with professional actors demonstrating the delivery of the BAP by a clinician with a patient; 4) clinical vignettes to apply the BAP using case scenarios; 5) a training workshop to practice and receive feedback when delivering the BAP; and 6) use of an opinion leader. The main roles of the opinion leader are to advise colleagues about SMS practice and ease the delivery of SMS.

Taking into account the teaching institution calendar year and curriculum, the KT intervention will be delivered as follows: clinicians and interns will first be asked to complete the self-study webinar and online educational module. They will then receive practice BAP and feedback from the opinion leaders. Clinicians and interns will also receive supportive materials on motivational interviewing and on how to deliver SMS guiding by BAP. They will also attend one- day training session delivered by a BAP trainer and have more opportunity to practice SMS and get personalised feedback. Further, 2 clinicians agreeing to act as champions (i.e. opinion leaders) will attend a BAP training to become certified in this approach prior to implementation the KT intervention. The main roles of the opinion leaders will be to support other clinicians and interns in using SMS and to provide them with coaching on applying the BAP with patients. Additional file 9 presents the final selection of KT intervention.

## Discussion

Organisational support increases the likelihood of clinicians’ successful uptake of EBP and CPG recommendations [90, 91]. Decision makers working at CMCC perceived that faculty and supervisory clinicians were highly committed to and confident about implementing SMS for patients with spine pain. Participating clinicians and interns showed positive attitudes toward EBP, and behaviours associated with EBP, which is consistent with the literature [92, 93]. These findings suggest that SMS strategies can be implemented in this environment.

Nonetheless, some barriers corresponding to four TDF domains that restricted both clinicians’ and interns’ use of SMS: *Knowledge, Skills, Environmental context and resources, and Emotion*. Aadditional barriers corresponding to three TDF domains that restricted the intern’ use of SMS were: *Beliefs about Capabilities; Memory, attention & decision making; and Social Influence*. To address these barriers, a panel of experts mapped BCTs to each barrier and selected the appropriate intervention components.

Both clinicians and interns felt that they needed more training to improve their knowledge and skills on the use of SMS, and they reported that lack of time was a key barrier to using SMS. Interns also indicated that they had a lack of confidence to use SMS. These findings are consistent with the literature showing that clinicians do not have sufficient knowledge and confidence in how to use SMS, and that they lack the appropriate training and competence to use SMS with patients [94, 95]. Furthermore, as the clinicians and interns did not receive intensive training on SMS, they admited sometimes feeling anxious about the use of SMS with complex patients. These findings are supported by the planned change theories, where the knowledge and skills are required to achieve confidece, [96], which may reduce the likelhood of anxiety [97]. In addition, according to these theories the presence of an opinion leader may improve one’s confidence regarding behavior change [96].

Not surprisingly, novices starting to develop their clinical judgment skills and working under the supervisory clinicians faced additional challenges in using SMS than clinicians. Interns indicated that they lacked the confidence and knowledge needed to routinely incorporate SMS, did not follow a systematic process to deliver SMS to patients, and had to rely on supervisory clinicians’ advice, even though some may not be comfortable or willing to use SMS in their own clinical practice. Together, these findings support the need to target both chiropractic interns and supervisory clinicians with strategies to help them improve their uptake and use of SMS in the clinical teaching environment.

Both clinicians and interns were generally motivated to use SMS in the clinical setting. This might be related to their beliefs about the effectiveness of SMS as well as to the collaborative nature of the relatiosnhips between clinicians and collegues, and the support from managers. In addition, the interns in this study stated that they would keep delivering SMS if it improved patients’ health outcomes. This is consistent with the operant learning theory where the achievements of a behaviour determines the continued use of that behaviour in the future [68].

The expert panel proposed different KT intervention strategies based on BCTs aimed at addressing the key barriers to using SMS among clinicians and interns. The selected KT intervention components formed a multifaceted theory-based intervention, which aims to simultaneously overcome several barriers [98]. The main KT intervention components were selected based on the current evidence [99] and feasibility to be implemented in the chiropractic clinical settings. These include supportive educational material, a webinar, an online educational module, a training workshop, and support by opinion leaders. A high-quality review demonstrated that implementing educational meetings, either alone or combined with other interventions, significantly improved the clinicians’ practice in the clinical setting [100]. Furthermore, two high-quality reviews showed that using educational material was effective for improving healthcare providers’ practice [101, 102]. Educational material could change clinicians’ beliefs, which may result in behaviour change among clinicians toward adherence to EBP [103]. In contrast, three other high-quality reviews showed that educational meetings had mixed effects for improving clinicians’ practice [104–107].

Of interest, the literature supported the effectiveness of internet-based learning (e.g. webinar, online module) on clinicians’ knowledge [108, 109]; internet-based learning had a larger positive effects than no intervention [109]. However, it had small effect comparing to non-internet learning [109]. Lastly, the literature supports the effectiveness of having an opinion leader, alone or combined with other interventions, to facilitate clinicians’ practice behaviour change [110, 111] and promote the adherence to EBP [112]. Interestingly, the availability of an opinion leader was proposed as a factor that made the new intervention implementation quicker [113]. Opinion leader has a small but worthy effect on clinicians behaviour change [114].

### Strengths/ limitations

To our knowledge this is the first study aimed at developing a theory-based intervention to support the use of SMS among chiropractors and interns within an educational setting. The KT intervention components in this study were developed based on behavioural change theories using a systematic approach with a panel of experts. This may increase the likelihood of successful use of SMS in the clinical setting. A limitation of this study is that the results cannot be generalized to all chiropractic clinics. While the inclusion of additional clinicians may have resulted in different views, barriers identified are similar to those found on the use of multimodal care by practicing chiropractors when managing neck pain [68].

## Conclusion

The key TDF factors that influence the uptake of SMS among clinicians and interns included: *knowledge, skill, environmental context and resources,* and *emotion.* Three additional TDF factors were identified only by interns: *Beliefs about Capabilities; Memory, attention & decision making;* and *Social Influence*. This may optimize the delivery of self-management support in spine pain clinics. The effectiveness of the selected KT intervention component remains to be tested.

## Additional files

Additional file 1: “Topic guide for focus groups with chiropractic interns and clinicians”. It provides the interview guide for focus group. (DOCX 14 kb)

Additional file 2: An Excel sheet that provides the raw data for clinicians and interns (XLSX 99 kb)

Additional file 3: An Excel sheet that provides the raw data for decision makers (XLSX 10 kb)

Additional file 4: “Thematic analysis based on the TDF – Clinicians”. It provides number of clinicians’ statements for each TDF domain, TDF specific beliefs and themes. (DOCX 17 kb)

Additional file 5: “Thematic analysis based on the TDF – Interns”. It provides number of clinicians’ statements for each TDF domain, TDF specific beliefs and themes. (DOCX 18 kb)

Additional file 6: “Specific Beliefs for each TDF with illustrative quotes – Clinicians”. It provides clinicians quotes representing specific TDF domains and beliefs. (DOCX 17 kb)

Additional file 7: “Specific Beliefs for each TDF with illustrative quotes – Interns”. It provides interns quotes representing specific TDF domains and beliefs. (DOCX 20 kb)

Additional file 8: “Mapping behaviour change techniques on key domains, proposed KT interventions and actions”. It provides a list of self-management-TDF barriers and the proposed KT interventions. (DOCX 19 kb)

Additional file 9: “Final Selection of KT Intervention Components and Related Learning”. It provides the final selection of KT intervention for clinicians and interns to promote the use of self-management support in the clinic. (DOCX 14 kb)

## Abbreviations

BAP: Brief action planning
BCT: Behavioural change technique
CMCC: Canadian Memorial Chiropractic College
CPGs: Clinical practice guidelines
EBP: Evidence-based practice
IQR: Interquartile range
KABQ: Knowledge, Attitude, and Behaviour Questionnaire
KT: Knowledge translation
ORIC: Organizational Readiness for Implementing Change
PABS: Pain Attitudes and Beliefs Scale
PAM: Patient activation measure
PMT: Patient Management Teams
SMS: Self-management strategy
TDF: Theoretical domain framework
