# Supplementary material for: Promoting the use of self-management in novice chiropractors treating individuals with spine pain: the design of a theory-based knowledge translation intervention
Source: BMC Musculoskelet Disord. 2018 Sep 11;19:328. doi: 10.1186/s12891-018-2241-1 (PMC6134709; doi:10.1186/s12891-018-2241-1)
Supplement: Supplementary file 1 — “Topic guide for focus groups with chiropractic interns and clinicians”. It provides the interview guide for focus group. (DOCX 14 kb) [file 12891_2018_2241_MOESM1_ESM.docx]

Additional File 1: Topic guide for focus groups with chiropractic interns and clinicians

I’d like to begin by asking you to state your name slowly for the benefit of the person who will be transcribing the interview.

Could you describe how you manage patients with back or neck pain in your office. By that I mean your usual routine once the patient is with you in the examining room, (such as case history questions), examination procedures, and whether managing acute or chronic back or neck pain patients using SMS strategy as part of the initial visit.

Prompts: What do you feel you do well? What do you feel you could do better?

Thank you.

Now for the rest of the interview, I have some slightly more specific questions. Some may seem repetitive, but please bear with me. We would like to understand how chiropractors make practice decisions about managing patients with neck pain. I may also seek clarification during the interview via probing questions such as: ‘What do you mean’; ‘Would you explain that’; ‘What were you thinking at the time’; ’Walk me through your experience’, ‘What skills do you need?’ ‘How and why do you use it?’ I would like to encourage you to say as much as you like, be as detailed and descriptive as you can.

**** For the purpose of this interview, ‘non-specific’ refers to patients with uncomplicated mechanical neck pain that varies with time and activity with no neurologic deficits, fractures or indicators of potentially serious pathologies (i.e. red flags)**

**Knowledge**

1. Tell me about the evidence surrounding optimal patient management using Self-Management

Support (SMS) for patients with non-specific back or neck pain?

(Prompt: Did reading the guidelines improve your knowledge?)

2. Do you adhere to any specific guideline to help you make informed decisions about when to use

SMS, including specific advice and home exercises, to patients with back or neck pain?

(Prompts: How do you use it? Why do you use it? What do you think of it?

3. Do you agree with the guideline contents?

(Prompt: What alternative, if any, would help you adopt the guideline’s recommendations?)

4. Are the guidelines representative of the evidence on SMS (quality, appropriateness)?

(Prompt: Do the guidelines reflect the evidence?)

**Beliefs about capabilities (self-efficacy)**

5. How confident are you in managing either acute and chronic non-specific back or neck pain using SMS?

(Prompt: How about education/advice? Exercises?),

(Prompts: How easy or difficult is this? What skills are required to manage patients with these conditions? What problems or barriers have you encountered in managing patients with back or neck pain?)

**Behavioural regulation**

6. Do you monitor changes in patients’ back or neck pain? Health status? After treating them using SMS? What is the usual outcome?

(Prompt: do patients improve/deteriorate?)?

7. What could help you manage back or neck pain patients using SMS?

8. Do you assess the patient’s motivation to follow advice and perform home exercises? If so, how? How do you manage a non-compliant patient?

**Skills**

9. What new skills would you require to be able to manage back or neck pain patients using SMS?

(Prompts: additional training, counselling skills /communication techniques, continuing education, educational material, online information).

10. What do you think about the importance of communication skills for the management of patients using SMS?

(Prompt: Why?)

11. What do you usually say or how do you manage patients who ask only for SMS but where you find it is not to their benefit?

**Intention**

12. On a scale where zero means ‘none of the time’ and five means ‘most of the time’, do you intend to manage non-specific back or neck pain patients using SMS?

(Prompts: Why or why not?)

**Goals**

13. What is the importance (i.e. priority) of providing SMS in the context of other tasks (such as delivering spinal manipulative therapy) in achieving the desired patient outcome?

(Prompt: Can you elaborate?)

**Memory, Attention and Decision Processes**

14. How easy or difficult is it to decide if a particular new patient needs SMS or not?

15. What rules of thumb, if any, do you use to reach a decision about providing SMS?

(Prompts: Decision rules, guidelines…)

**Reinforcement**

16. On a scale where zero means ‘none of the time’ and five means ‘most of the time’’, would you manage back or neck pain patients using SMS if the rewards were greater than when using manual therapy alone?

(Prompts: Better patient satisfaction, less patient discomfort, etc.)

**Beliefs about consequences (Anticipated outcome/attitude)**

17. What are the benefits of managing a patient using SMS for either acute or chronic non-specific back or neck pain?

(Prompt: Better patient health outcomes? Shorter recovery times?)

18. What are the potential disadvantages of managing patients with non-specific back or neck pain without offering proper SMS?

(Prompts: 1) longer recovery time 2) patient preference and satisfaction)

19. How do you perceive the safety of SMS? Have these guidelines changed your impressions?

**Environmental context and resources (environmental constraints)**

20. Are there any factors in your practice likely to either help or prevent you from using SMS with patients with non-specific back or neck pain?

(Prompts: Do you think having or not having visual support (e.g., pamphlet, video, online module) influences your decision to manage non-specific back or neck pain patients using

SMS?

21. Does running late or having a staff shortage on any particular day influence whether or not you decide to use SMS when managing non-specific back or neck pain patients?

Are there any resources available that you use to help you manage non-specific back or neck pain patients using SMS?

(Prompts: Information pamphlets or posters to inform patients about the benefits of regular exercise, healthy eating or other life style changes)

**Social influences (Norms)**

22. Are there instances where you may consider consulting other people for their opinion regarding the need for providing SMS?

(Prompts: Peers, managers, other professional groups, patients)

23. How do the views of other colleagues or organizations influence your decision to offer SMS to your patients?

(Prompts: Do you interact with chiropractic opinion leaders in your province?)

**Emotion (Stress/concerns)**

24. How do you respond to distressed patients?

25. Is seeing acute back or neck patients in apparent distress likely to influence your decision to use

SMS?

26. Does seeing a patient with chronic back or neck pain, particularly if there is a psychological overlay such as depression, influence your decision in terms of how you manage that patient with respect to using SMS?

**Social/professional role and identity (self-standards)**

27. Do you think it is appropriate that your role should include managing patients with back or neck pain using SMS?

Those are all of the questions I have for you today. Has anything else occurred to you about this topic that we haven’t asked about? Are there any other thoughts you wish to convey on this topic?
