# Supplementary material for: Promoting the use of self-management in novice chiropractors treating individuals with spine pain: the design of a theory-based knowledge translation intervention
Source: BMC Musculoskelet Disord. 2018 Sep 11;19:328. doi: 10.1186/s12891-018-2241-1 (PMC6134709; doi:10.1186/s12891-018-2241-1)
Supplement: Supplementary file 4 — “Thematic analysis based on the TDF – Clinicians”. It provides number of clinicians’ statements for each TDF domain, TDF specific beliefs and themes. (DOCX 17 kb) [file 12891_2018_2241_MOESM4_ESM.docx]

Additional file 4: Thematic analysis based on the TDF – Clinicians

| TDF domain | Questions (N) | Utterances (N) | Specific beliefs (N) | Specific beliefs (number of utterances) | Increase N (%) | Decrease N (%) | No Influence N (%) | Themes |
| --- | --- | --- | --- | --- | --- | --- | --- | --- |
| Knowledge | 2 | 16 | 3 | - I am aware of the evidence and guideline for using SMS for patients with spine disorders **(N = 4)** - We gained knowledge of SMS from different courses/ no specific course for SMS **(N = 6)** / course on SMS needed **(N = 1)** - Student/clinician lack the knowledge of using SMS **(N = 3)** / not lack **(N = 2)** | 6 (37%) | 10 (63%) |  | Awareness of the guidelines; SMS knowledge |
| Skills | 2 | 12 | 2 | - Students have skills of using SMS **(N = 6)**, other HCPs don’t have sufficient skills of using SMS **(N = 1),** Chiropractors don’t have sufficient skills of using SMS **(N = 1)** - Course needed to gain SMS skills **(N = 2)**, Counselling and communication skills needed **(N = 2)** | 6 (50%) | 5 (42%) | 1 (8%) | Skills needed to use SMS |
| Social Professional Roles | 2 | 25 | 4 | - Managing spine pain patient using SMS is a part of my role as a chiropractor **(N=14),** SMS isn’t not a part of my role **(N = 5),** SMS could/couldn’t be part of HCP role **(N = 1)** - Making appropriate referral to other HCPs is a part of my role **(N = 2)** - Chiropractors should know their SOP and when to refer on **(N = 1)** / I use guidelines in my decision-making **(N = 1)** - Being respectful towards other professions is a part of my role **(N = 1)** | 19 (76%) | 5 (20%) | 1 (4%) | Professional role (SMS is a part of chiropractic role) |
| Beliefs about Capabilities | 2 | 29 | 4 | - I am very confident /somewhat confident in managing spine pain using SMS **(N= 5)** - I am comfortable in managing spine pain using SMS **(N= 2)** / new grads are not comfortable in using SMS **(N = 1)** - Having ability to deliver SMS **(N = 15**) / ability is limited **(N = 3)** - Not easy to deliver SMS **(N = 3)** | 22 (76%) | 7 (24%) | 0 | Acceptance, capabilities |
| Optimism | - | - | - | - | - | - | - | - |
| Beliefs about Consequences | 2 | 20 | 3 | - Benefits of SMS include: better patient outcome, increase confidence, financial benefit, improve QOL, decrease pain, decrease psychological symptoms, ability to perform activities **(N = 16)** - Disadvantages of SMS include: spending time among clinicians, negative financial influence for clinicians **(N = 2)** / non proper advice may lead to immense consequence **(N = 1)** - Not using SMS decreases the influence of the profession with other healthcare providers **(N = 1)** | 16 (80%) | 3 (15%) | 1 (5%) | Consequence of managing spine pain patients with SMS |
| Reinforcement | 1 | 2 | 2 | - I would definitely manage spine disorders with SMS if I knew the rewards were greater **(N = 1)** - Interns need to be encouraged to use SMS with patients **(N = 1)** | 2 (100%) | 0 | 0 | Better outcomes reinforce use SMS |
| Intention | 1 | 8 | 1 | - I will use SMS all the time / a lot **(N = 8)** | 8 (100%) | 0 | 0 | Decision to manage patients using SMS |
| Goals | 1 | 16 | 3 | - SMS is a high priority **(N = 2)** / SMS is not a priority **(N = 1)** - SMS is an important treatment **(N = 3)** / important as other treatment **(N = 1)/** the importance of SMS varies according to the condition of patient **(N = 1)** - Our goal is to empower patient **(N = 7)/** to implement SMS **(N = 1)** | 14 (88%) | 1 (6%) | 1 (6%) | Importance and the priority of SMS |
| Memory, attention & decision making | 1 | 12 | 4 | - The decision making on SMS components depends on patients’ needs **(N = 6)** - I use evidence to guide my decision on the use of SMS **(N = 2)** / I use my own intuition to guide my decisions on the use of SMS **(N = 1)** / Interns need to understand that guidelines on SMS must be applied using clinical judgment and patient preference **(N = 1)** - I decide to refer my patients if they have psychological overlay **(N = 1)** - I sometimes forget to provide complete information to patients **(N = 1)** | 9 (75%) | 3 (25%) | 0 | The decision means on the use of SMS |
| Environmental and context resources | 2 | 31 | 4 | - Having good time management helps me use SMS **(N = 4)** / I have enough time to use SMS **(N = 1)/** lack of time is a barrier to use SMS **(N = 5)** - Educational Material on exercise would help implement SMS **(N=3)** - Clinic characteristics that **facilitates** the use of SMS: having rehab equipment and sufficient space **(N = 2),** clinician characteristics (collaborative) **(N = 2),** having interns on placement **(N = 1)** - Patient characteristics that **restrict** the use of SMS: Patient's lack of compliance, resources, and/or time **(N = 6)**, patients priorities **(N = 2)**, depression **(N = 2),** not accepting the condition **(N = 1)**, not trust the clinicians **(N = 1),** language and cultural barriers **(N = 1)** | 13 (42%) | 18 (58%) | 0 | Environmental factors of using SMS:  Time, clinic characteristic, patient characteristic |
| Social Influence | 2 | 8 | 2 | - There are instances (including cultural barrier) when I would consult other people on using SMS **(N = 3)** / I enjoy asking and learning from other clinicians **(N = 2)** - Patient status/behavior makes me deliver SMS **(N = 1)/** Patient status/behavior restricts me from delivering SMS **(N = 2)** | 6 (75%) | 2 (25%) | 0 | Influence of others (colleagues, patients) |
| Emotion | 2 | 11 | 3 | - I feel excited/great about using SMS **(N = 3)** - We feel anxious when we use SMS with some patients (especially who have psychological overlay) **(N = 5)** / New graduates could feel anxious about their ability to use SMS **(N = 1)** - I am terrified that self-management guideline or roadmap will discourage students from using their clinical judgement **(N = 1)** / I am worried about lack of specificity in guidelines because it can result in decreased efficacy **(N = 1)** | 3 (27%) | 7 (64%) | 1 (9%) | Feeling anxiety on the use of SMS |
| Behavioral Regulation | 3 | 21 | 3 | - I assess patient motivation toward SMS **(N = 3)** / I use the Report of Findings to understand patient's motivation **(N = 2)/** I misjudge the level of motivation of a patient **(N = 1)/** I have to figure out how to motivate patients toward SMS **(N = 1).** - I adapt SMS for each patient individually **(N = 10)** / If a patient is catastrophizing you may have to change the approach of using SMS **(N = 1)** - My routine clinical practice includes use SMS **(N = 1)/** I manage my time to implement SMS **(N = 2)** | 20 (95%) | 1 (5%) | 0 | Assessing patient motivation toward SMS, clinical practice of SMS (adapted to the patient needs) |
