# Supplementary material for: Promoting the use of self-management in novice chiropractors treating individuals with spine pain: the design of a theory-based knowledge translation intervention
Source: BMC Musculoskelet Disord. 2018 Sep 11;19:328. doi: 10.1186/s12891-018-2241-1 (PMC6134709; doi:10.1186/s12891-018-2241-1)
Supplement: Supplementary file 5 — “Thematic analysis based on the TDF – Interns”. It provides number of clinicians’ statements for each TDF domain, TDF specific beliefs and themes. (DOCX 18 kb) [file 12891_2018_2241_MOESM5_ESM.docx]

Additional file 5: Thematic analysis based on the TDF – Interns

| TDF domain | Questions (N) | Utterances (N) | Specific beliefs (N) | Specific beliefs (number of utterances) | Increase N (%) | Decrease N (%) | No Influence N (%) | Themes |
| --- | --- | --- | --- | --- | --- | --- | --- | --- |
| Knowledge | 2 | 45 | 3 | - I am aware (or partially aware) of the evidence and guideline for using SMS for patients with spine disorders **(N = 18)** / not aware **(N = 3),** guideline is Vague **(N = 2)** - Having knowledge of using SMS **(N = 10)**, not sure of how to use SMS **(N = 4),** other HCPs don’t have sufficient knowledge of using SMS **(N = 1)** - Interns gained knowledge about SMS from other chiropractor **(N = 2)/** needed courses for SMS **(N = 5)** | 30 (67%) | 14 (31%) | 1 (2%) | Awareness of the guidelines; SMS knowledge |
| Skills | 2 | 41 | 3 | - Having skill of using SMS **(N = 6)/** Lack of skills of using SMS **(N = 9)** - Receiving training of using **SMS (N = 2)**/ limited training **(N = 1),** not receiving training of using SMS including CBT **(N = 7)** - Lack of behavioral change skills **(N = 3)** - Training courses needed to gain more skills of using SMS **(N = 9)** | 8 (20%) | 33 (80%) |  | Lack of skills and training of using SMS |
| Social Professional Roles | 2 | 45 | 5 | - Managing patients with spine pain using SMS is a part of my role as a chiropractor **(N=29),** not part of my role **(N = 1)** - Spinal manipulative Therapy (SMT) is my primary role to manage patients with spine pain **(N = 3)** - Students being pressurized by faculty/clinicians to prioritize SMT as a primary treatment **(N = 4),** not being pressurized **(N = 1)** - We refer some patients to other HCPs for SMS **(N = 6)** - It is important to choose to protect my professional and personal boundaries **(N = 2)** | 30 (67%) | 13 (29%) | 2 (4%) | Professional role (SMS is a part of the chiropractors role) |
| Beliefs about Capabilities | 2 | 52 | 6 | - I am confident /somewhat confident in managing spine pain using SMS **(N= 14)** / not confident **(N = 2)** - I am fairly comfortable in managing spine pain using SMS **(N= 2)**/ our level of comfort with SMS depends on our ability to demonstrate exercises **(N = 1)/** I feel uncomfortable when I am not sure if a patient is capable of completing an exercise or not **(N = 1)** - Having the ability to deliver SMS **(N = 13)** / ability is limited **(N = 3)** - Not easy to deliver SMS with some patients **(N = 10)** - Factors increase confidence include: see patients benefiting from SMS **(N = 1)**, having experience with SMS **(N = 4),** asking clinicians and colleagues **(N = 1)** - It is important to show the patient that you are confident in what you are doing **(N = 1)** | 29 (56%) | 16 (31%) | 7 (13%) | Acceptance, capabilities |
| Optimism | - | 15 | 1 | - Optimistic about using SMS **(N = 12)/** not optimistic about using SMS **(N = 3)** | 12 (80%) | 3 (20%) | 0 | Optimism of using SMS |
| Beliefs about Consequences | 2 | 44 | 2 | - Benefits of SMS include: better patient outcome, increase confidence, empower patients, decrease psychological symptoms, ability to perform activities, prevent further injury, more efficient patient recovery, wellness, save people time and community/clinic resources **(N = 31)/** non proper SMS aggravates problem and tarnish the reputation of the profession **(N = 2)** / disadvantage of not motivating in SMS include suboptimal health outcomes **(N = 1)/** disadvantage of **not** using SMS include: make patients dependent on passive care, make patients get wrong information, make patients spend more money on prescription medication **(N = 9)** - When you see patients benefiting from SMS it makes you more confident as a doctor **(N = 1)** | 43 (98%) | 1 (2%) | 0 | Consequence of managing spine pain patients with SMS |
| Reinforcement | 1 | 16 | 3 | - I would definitely manage spine disorders with SMS if I knew the rewards were greater **(N = 10)/** I would not use SMS exclusively if I knew the benefits were better than manual therapy alone **(N = 2)** - Passive/non-motivated patients don’t reinforce me to implement SMS **(N = 2)/** Learning SMS by non-experienced doesn’t reinforce me to deliver SMS **(N = 1)** - Chiropractic organizations could encourage clinicians to use SMS **(N = 1)** | 11 (69%) | 3 (19%) | 2 (13%) | Better outcomes reinforce use SMS |
| Intention | 1 | 13 | 2 | - I will use SMS all the time / a lot **(N = 12)** - The decision to use SMS depends on whether the chiropractor wants to use it or not **(N = 1)** | 12 (92%) | 0 | 1 (12%) | Decision to manage patients using SMS |
| Goals | 1 | 15 | 3 | - SMS is a priority treatment **(N = 5)** / SMS is prioritized as SMT **(N = 1)** - SMS is an important treatment **(N = 3)** / more important than SMT **(N = 2)/** important as SMT **(N = 2)** - Our goal is to empower patient **(N = 2)** | 15 (100%) | 0 | 0 | The priority of SMS |
| Memory, attention & decision making | 1 | 23 | 5 | - The decision making on SMS components depends on patients’ needs **(N = 7)** - We don’t use guidelines to guide the decision on the use of SMS **(N = 2)/** we use clinical judgment to decide to use SMS **(N = 2)/** I use my intuition to decide to use SMS **(N = 1)** - I decide to use SMS before passive care **(N = 6)** - I decide to use passive care to keep my patients **(N = 1)** - I decide to refer my patients if they have psychological overlay **(N = 4)** | 13 (57%) | 10 (43%) | 0 | The decision means on use of SMS |
| Environmental context resources | 2 | 70 | 8 | - Lack of time is a barrier to use SMS **(N = 7)/** using SMS save time for long term **(N = 1)/** lack of time makes me use only SMS **(N = 1)/** lack of time makes me use only SMT **(N = 1)** - Clinic characteristics that **facilitate** the use of SMS: clinician characteristics (collaborative) **(N = 3),** having kinesiology students **(N = 2),** smaller case load **(N = 1)**   Clinic characteristics that **restrict** the use of SMS: Lack of space and equipment **(N = 4),** staff shortage **(N = 3)**, clinician characteristics (unaware of guidelines) **(N = 1),** lack of communication with peers **(N = 1),** I did not have enough exposure to different patient conditions **(N = 1)/** our clinicians should teach us how to use SMS **(N = 1)**   - Financial consideration is a barrier to use SMS **(N = 7)** - The internship course requirements are a barrier to my use of SMS **(N = 8)** - Patient characteristics that **restrict** the use of SMS: fear avoidance behavior **(N = 3),** obstacles that patients face can vary **(N = 1),** patient adherence to SMS **(N = 2),** Patient motivation to use SMS **(N = 8),** patient preferences **(N = 1)** - Educational Material contributes to patient adherence to SMS **(N = 10)** - We lack comprehensive guidelines suggesting SMS **(N = 1)** - More focused rehab class would be helpful for interns **(N = 1)/** SMS ought to be integrated earlier in students' education **(N = 1)** | 17 (24%) | 50 (71%) | 3 (4%) | Environmental factors of using SMS: time, clinic characteristics, patient characteristics, financial issues, lack of guideline, course training |
| Social Influence | 2 | 34 | 4 | - The views of my clinician influence my use of SMS: restrict **(N = 8),** facilitate **(N = 6),** SO-SO **(N = 1)** / I consult my supervisory clinician on my use of SMS **(N = 5)** and colleagues **(N = 3)** on my use of SMS/ Clinician preference affects student utilization of SMS **(N = 1)** - Patient attitude (preferring passive care or highly motivated) restrict the use of SMS **(N = 6)/** patient acceptance and readiness for SMS affect my SMS practice **(N= 1)** - SMS should be taught by people who actually believe in it **(N = 2)** - Institutional practices influence my perception of SMS **(N = 1)** | 14 (41%) | 16 (47%) | 4 (12%) | Influence of others (supervisors, colleagues, patients) |
| Emotion | 2 | 33 | 5 | - I feel disappointed because of clinicians behaviours: prioritizing one treatment over another, non-aware of the guideline, don’t use SMS **(N = 4)** / I feel stressed because of the internship course requirements don’t include SMS assessment **(N = 1)** - I feel excited/good/optimistic about using SMS **(N = 6)** / I feel anxious/nervous when use SMS **(N = 3)/** I feel concerned/frustrated when patient don’t adhere to SMS **(N = 7)** - I feel optimistic/motivated when I see patients benefitting from SMS **(N = 5)/** I feel hopeful when patient improved because of SMS **(N = 1)** / I feel hopeless when patient not improved or have psychological overlay **(N = 4)** - I feel nervous about referring on to kinesiology students because patients may think that SMS is not important **(N = 1)** - My emotion status determines my treatment approach **(N = 1)** | 13 (39%) | 19 (58%) | 1 (3%) | Emotion toward use SMS |
| Behavioral Regulation | 3 | 63 | 5 | - I adapt SMS for each patient individually **(N = 25)/** I monitor changes in patient's condition and change SMS components according to patient achievement **(N = 2)** - Providing feedback to patients on their progress between visits helps to motivate them **(N = 8)/** Providing feedback to patients on their progress during visits could be demotivating if they don't improve **(N = 2)/** providing feedback to patients on their progress between visits helps to motivate them **(N = 2)/** Assessing patients readiness for SMS is important to success it **(N = 1)/** patient motivation is needed **(N = 2)** - SMS is a routine clinical practice **(N = 4)** - Using visual cues to reinforce patient increase the adherence to SMS **(N = 6)/** breaking down the SMS components facilitates SMS implementation **(N = 3)/** I will address the patient barrier to uptake SMS **(N = 1)/** we should assess patient SMS uptake **(N = 1)**/ designing a program to increase patient take of SMS **(N = 2)** - Patient education needs to be integrated in SMS **(N = 1)** | 61 (97%) | 2 (3%) |  | Assessing patient motivation toward SMS, adherence to SMS, clinical practice of SMS (adapted to the patient needs) |
