# Supplementary material for: Promoting the use of self-management in novice chiropractors treating individuals with spine pain: the design of a theory-based knowledge translation intervention
Source: BMC Musculoskelet Disord. 2018 Sep 11;19:328. doi: 10.1186/s12891-018-2241-1 (PMC6134709; doi:10.1186/s12891-018-2241-1)
Supplement: Supplementary file 6 — “Specific Beliefs for each TDF with illustrative quotes – Clinicians”. It provides clinicians quotes representing specific TDF domains and beliefs. (DOCX 17 kb) [file 12891_2018_2241_MOESM6_ESM.docx]

Additional file 6: Specific beliefs for each TDF with illustrative quotes – Clinicians

| **TDF domain** | **Specific beliefs** | **Selected Statements by participants** |
| --- | --- | --- |
| Knowledge | I am aware of the evidence and guideline for using SMS for patients with spine disorders | *“Empirical evidence. Anecdotal evidence. Yeah like we pick the guidelines from the evidence that applies to our patients.”* |
|  | We gained knowledge of SMS from different courses/ no specific course for SMS / course on SMS needed | *“there were various things that I think we pick up in different classes that…It wasn't all centralized into one, but I think it was maybe a variety”* |
|  | Student/clinician lack the knowledge of using SMS/ not lack | *“I think the students get a bit more of that now as they go through. We might not have had that”* |
| Skills | Students have skills of using SMS | *“I think also the idea of delivering messages, those are really good and I think they're getting trained now on the undergraduate on history taking*  *and how to elicit change from the patients”* |
|  | Course needed to gain SMS skills/ Counselling and communication skills needed | *“The easy answer is 'If you had a course in self-management, you would take that and you'd be perfect'. But if not, you're basically trying to put together an understanding of a few different things.”* |
| Social Professional Roles | Managing spine pain patient using SMS is a part of my role as a chiropractor/ SMS isn’t not a part of my role | *“You're going make that part of your care now. You do this. You're doing your job and I'm doing mine. You see how this works. This is the point*  *I'm trying to make, this is where we need to be, this is what we have to do to go forward.”* |
|  | Making appropriate referral to other HCPs is a part of my role | *“But I think we all sort of get an understanding of where these issues play into the MSK part of it. And we all want to help and we have some*  *ability to do that, but it's truncated ability. And so you do what you can do within the context of yourself”* |
| Beliefs about Capabilities | I am very confident /somewhat confident in managing spine pain using SMS | *“I'd say very confident”* |
|  | I am comfortable in managing spine pain using SMS/ new grads are not comfortable in using SMS | *“Yes very (comfortable).*  *“I think we're relatively confidence (yes). I could see maybe like a new grad who's not seen enough cases get a question and they're uncomfortable and they may not know the answer”* |
|  | Having ability to deliver SMS/ ability is limited | *“but I do hold myself accountable to the fact that everyone will leave my office knowing how to cope with their pain. It may be exercises, it may be knowing that they're not dying, it may be that they use ice or heat or whatever they love, it may be to stand up every 20 minutes, they're going to leave with something”* |
|  | Not easy to deliver SMS | *“It’s really complicated self-management for someone who doesn’t want to get out of bed to do something”* |
| Beliefs about Consequences | Benefits of SMS include: better patient outcome, increase confidence, financial benefit, improve QOL, decrease pain, decrease psychological symptoms, ability to perform activities | *“I think we know that SMS have a lot of positive benefits to patients”* |
|  | Disadvantages of SMS include: spending time among clinicians, negative financial influence for clinicians/ non proper advice may lead to immense consequence | *“The converse of all that Patient dependence (yeah) Potentially reinjure themselves or worsen their condition (yeah) Decreased compliance to our care for sure They may be more compliant to passive care”* |
| Reinforcement | I would definitely manage spine disorders with SMS if I knew the rewards were greater | *“Great extent! We already know that.”* |
|  | Interns need to be encouraged to use SMS with patients | *“They feel they are treating but the messages that go with the patient out the door probably take a little bit more encouragement because it doesn't come naturally right away”* |
| Intention | I will use SMS all the time / a lot | *“I definitely give self-care to all of them”* |
| Goals | SMS is a high priority/ SMS is not a priority | *“it's almost my number one priority. Because I don't necessarily adjust everyone.”* |
|  | SMS is an important treatment / important as other treatment**/** the importance of SMS varies according to the condition of patient | *“So I think it's important to kind of sit back and have a chat again”* |
|  | Our goal is to empower patient**/** to implement SMS | *“you're helping them get ahead of the problem”* |
| Memory, attention & decision making | The decision making on SMS components depends on patients’ needs | *“And recognizing those things on an individual basis is key, because you just can't cut it out. It's not a cookie cutter approach. It's individual.”* |
|  | I use evidence to guide my decision on the use of SMS/ I use my own intuition to guide my decisions on the use of SMS/ Interns need to understand that guidelines on SMS must be applied using clinical judgment and patient preference | *“Well you know what, I think it comes back to what evidence-based medicine is. You have your three intersecting pillars you know those diagrams. Evidence, right, and then your patient preferences and then our experience. I think that's how we all kind of train and try to make our best decisions.”* |
| Environmental and context resources | Having good time management helps me use SMS / I have enough time to use SMS**/** lack of time is a barrier to use SMS | *“When they have less time, or they don't understand the exercise, maybe you might spend a little bit less time showing them the exercise. Why don't we review this when you're back in a couple of days? I'm more likely to do something like that maybe.”* |
|  | Educational Material on exercise would help implement SMS | *“if they still didn't get it then there was no sense giving them a whole sheet of paper to do because it was going to be lost anyway. I still do the handouts or emails sometimes, but what I found kind of works a little bit now is I'll use their own phone and record them.”* |
|  | Clinic characteristics that **facilitates** the use of SMS: having rehab equipment and sufficient space, clinician characteristics (collaborative), having interns on placement | *“We’re all very open, very willing to learn. So again I know we’re only taking a slice of CMCC. I think this is a very unique slice of the chiropractic pie”* |
|  | Patient characteristics that **restrict** the use of SMS: Patient's lack of compliance, resources, and/or time, patients priorities, depression, not accepting the condition, not trust the clinicians, language and cultural barriers | *“But if you have someone’s who’s expressing depression and distress and they’re catastrophizing, they’re not really uptaking the information, certainly that’s a barrier to them doing this“* |
| Social Influence | There are instances (including cultural barrier) when I would consult other people on using SMS/ I enjoy asking and learning from other clinicians | *“Yes, I think so. I might ask my colleague here or someone else. In my own private practice we have other practitioners and sometimes have little tidbits that I wasn't aware of, and like, 'aw that's cool' I'm going to try that next time.”* |
|  | Patient status/behavior makes me deliver SMS**/** Patient status/behavior restricts me from delivering SMS | *“I think I recall not really knowing how important advice was maybe when I first started, and then you’re faced with having to give advice by force really because your patients are asking you questions on what I can and cannot do”* |
| Emotion | I feel excited/great about using SMS | *“Yep I feel great about it”* |
|  | We feel anxious when we use SMS with some patients (especially who have psychological overlay) / New graduates could feel anxious about their ability to use SMS | *“You can feel pretty anxious as well I think too, (yes) you're not sure how much…”* |
| Behavioral Regulation | I assess patient motivation toward SMS/ I use the Report of Findings to understand patient's motivation**/** I misjudge the level of motivation of a patient**/** I have to figure out how to motivate patients toward SMS | *“I get a pretty good sense if someone’s going to listen to me or do what’s prescribed. I don’t know if I do it directly. I kind of garner from speaking to the patient and seeing how receptive they are to what we’re talking about and stuff like that”* |
|  | I adapt SMS for each patient individually/ If a patient is catastrophizing you may have to change the approach of using SMS | *“in real life, most people, especially if they're self-employed, if they're not working, they're not getting paid, so you have to be able to get them*  *back, so you have to still give them strategies to deal with it over the course of the day. And once they can get their demand under control you can build up their capacity again to get past that level, and then you can kind of move things up from that new baseline”* |
|  | My routine clinical practice includes use SMS**/** I manage my time to implement SMS | *“I think you would know what the most commons are maybe for certain groups of people, so you still have most commons that you know you’re going to go to, so if I was to say I had probably not a procedure, but a routine maybe”* |
