# Supplementary material for: Promoting the use of self-management in novice chiropractors treating individuals with spine pain: the design of a theory-based knowledge translation intervention
Source: BMC Musculoskelet Disord. 2018 Sep 11;19:328. doi: 10.1186/s12891-018-2241-1 (PMC6134709; doi:10.1186/s12891-018-2241-1)
Supplement: Supplementary file 7 — “Specific Beliefs for each TDF with illustrative quotes – Interns”. It provides interns quotes representing specific TDF domains and beliefs. (DOCX 20 kb) [file 12891_2018_2241_MOESM7_ESM.docx]

Additional file 7: Specific beliefs for each TDF with illustrative quotes – Intern

| TDF domain | Specific beliefs (number of utterances) | Selected Statements by participants |
| --- | --- | --- |
| Knowledge | I am aware (or partially aware) of the evidence and guideline for using SMS for patients with spine disorders/ not aware/ guideline is Vague | *“I think it might be good especially since our school has been drilling guidelines into us”* |
|  | Having knowledge of using SMS, not sure of how to use SMS | *“So there are not any major guidelines to it. It'll say okay identify this and do this but like it doesn't actually give you a specific way of handling a situation so it's kind of vague. And you kind of have to go in and hope for the best so…and maybe call it CBT.”* |
|  | Interns gained knowledge about SMS from other chiropractor**/** needed courses for SMS | *“There should almost be a course (in SMS)”* |
| Skills | Having skill of using SMS**/** Lack of skills of using SMS | *“The stages of change model. I feel that we've learned it textbook. But we haven't actually applied it. And until we apply it, we really don't know what we're doing. And I find that most of my learning has come from the actual experience in clinic.”* |
|  | Receiving training of using SMS/ limited training, not receiving training of using SMS including CBT | *“The actual skill though of delivering the information, you get no training on.”* |
|  | Lack of behavioral change skills | *“The stages of change model. I feel that we've learned it textbook. But we haven't actually applied it. And until we apply it, we really don't know what we're doing. And I find that most of my learning has come from the actual experience in clinic.”* |
|  | Training courses needed to gain more skills of using SMS | *“we have the knowledge to educate, but we don't necessarily have the charisma or the personality skills or the social skills as a doctor to empower a patient. And I think those are two very different and two very, very important aspects”* |
| Social Professional Roles | Managing patients with spine pain using SMS is a part of my role as a chiropractor, not part of my role | *“I think everyone needs educated in it. I think if you have good knowledge and you're willing to educate your patients, no matter what profession you are, I think you should. And this school does a good job teaching us multimodal care. And I think that should definitely be incorporated.”* |
|  | Spinal manipulative Therapy (SMT) is my primary role to manage patients with spine pain | *“I agree with that. You do have to make it business. But this is where I think I always have an issue with the chiropractic profession. Because they're obsessed with their own identity as 'the adjusters'.”* |
|  | Students being pressurized by faculty/clinicians to prioritize SMT as a primary treatment, not being pressurized | *“This question brings into light some bureaucracy surrounding our profession. As I mentioned before we were pressured by faculty to prioritize spinal manipulation as our primary mode of treatment. That's a huge source of bias in my opinion. And scientifically it's not literate at all.”* |
|  | We refer some patients to other HCPs for SMS | *“Some things like CBT I know a lot of chiros refer out. It's important to have that multimodal care.”* |
| Beliefs about Capabilities | I am confident /somewhat confident in managing spine pain using SMS/ not confident | *“I'm confident intervening with SM tools, usually.”* |
|  | I am fairly comfortable in managing spine pain using SMS/ our level of comfort with SMS depends on our ability to demonstrate exercises**/** I feel uncomfortable when I am not sure if a patient is capable of completing an exercise or not | *“I'm fairly comfortable in activity intervention, active care modalities. I'm probably less familiar with modification of ADLs”* |
|  | Having the ability to deliver SMS/ ability is limited | *“I just want to take us away from just exercise for a second. But we've also like in psychology we've learned about you know the steps of making a change and identifying where the patient is, in order to get a change from them.”* |
|  | Not easy to deliver SMS with some patients | *“Because if we’re trying to individualize the treatment, it’s hard to just provide rehab programs the way they are available out there with regards to guidelines”* |
|  | Factors increase confidence include: see patients benefiting from SMS, having experience with SMS, asking clinicians and colleagues | *“And I think that comfort where I don’t necessarily always have to do that but maybe with a little more complex things, sometimes it’s just nice to have that second opinion or third opinion to say what you’re doing is right”* |
| Beliefs about Consequences | Benefits of SMS include: better patient outcome, increase confidence, empower patients, decrease psychological symptoms, ability to perform activities, prevent further injury, more efficient patient recovery, wellness, save people time and community/clinic resources**/** non proper SMS aggravates problem and tarnish the reputation of the profession/ disadvantage of not motivating in SMS include suboptimal health outcomes**/** disadvantage of **not** using SMS include: make patients dependent on passive care, make patients get wrong information, make patients spend more money on prescription medication | *“So a big component of our SM was managing their anxiety, their moods, their fears about just going through daily life with those conditions chronic and acute”* |
| Optimism | Optimistic about using SMS**/** not optimistic about using SMS | *“It makes me feel that I've done a good job. I've done my due diligence as a future doctor, as an intern, as a support system for that patient. It makes me feel that I've hit the nail on the head. Given them something that they haven't gotten anywhere else.”* |
| Reinforcement | I would definitely manage spine disorders with SMS if I knew the rewards were greater**/** I would not use SMS exclusively if I knew the benefits were better than manual therapy alone | *“Not exclusively. I'll use my hands to some degree but I think it's much more powerful within session and then therefore between sessions. I see much more benefit out of giving them exercise. I'm always thinking exercise rather than other things just because that's what I'm always giving but I think it's very powerful.”* |
|  | Passive/non-motivated patients don’t reinforce me to implement SMS**/** Learning SMS by non-experienced doesn’t reinforce me to deliver SMS | *“That's the overarching thing. You have to empower them. And whether or not they're willing to empower themselves, or if they're just passively looking for you to take them out of pain. That's not a good situation to be in.”* |
| Intention | I will use SMS all the time/ a lot | *“Yeah, I think in an ideal world it would be all the time* (using SMS)*, every single patient. As much as possible”* |
| Goals | SMS is a priority treatment/ SMS is prioritized as SMT | *“It's a top priority. But financially… it can't be.”* |
|  | SMS is an important treatment/ more important than SMT**/** important as SMT | *“Always important and like crucial in managing like any spinal condition. So that’s what I’ve noticed”* |
|  | Our goal is to empower patient | *“Yeah I think you've got to definitely make it matter to them.”* |
| Memory, attention & decision making | The decision making on SMS components depends on patients’ needs | *“It's all dependent on who's in front of you, based on their lifestyle, what their goals are. You kind of take from the different areas, whether it be psychosocial, rehab, something else. It's all dependent on the patient.”* |
|  | We don’t use guidelines to guide the decision on the use of SMS**/** we use clinical judgment to decide to use SMS**/** I use my intuition to decide to use SMS | *“I think it's intuition”* |
|  | I decide to use SMS before passive care | *“What I do is I do active care before I do any passive care. So I get that done right out of the way, so you usually what ends up being shortchanged is the passive care.”* |
|  | I decide to refer my patients if they have psychological overlay | *“Yes not even just our colleagues but if you have a patient with several yellow flags or that has major depression, something that we can’t actually deal with, that we think our treatment plan could benefit and they could also benefit from co-management with either a psychologist, mental health professional, I think it’s really important to build those connections.”* |
| Environmental and context resources | Lack of time is a barrier to use SMS**/** using SMS save time for long term**/** lack of time makes me use only SMS**/** lack of time makes me use only SMT | *“I would say so there's not really any constraints to using SM but I would just say time but that's maybe just because I'm not efficient.”* |
|  | Clinic characteristics that **facilitate** the use of SMS: clinician characteristics (collaborative), having kinesiology students, smaller case load | *“So I think it's key that we have someone that's able to provide an accurate and proper diagnosis and then give SMS that don't harm the patient.”* |
|  | Clinic characteristics that **restrict** the use of SMS: Lack of space and equipment, staff shortage, clinician characteristics (unaware of guidelines), lack of communication with peers, I did not have enough exposure to different patient conditions**/** our clinicians should teach us how to use SMS | *“Equipment that's available has a huge…because in Sherbourne we had this really handy thing that one of the clinician's made. It was 60 degrees so you could do core strengthening and it was an easy tool to have, and you knew exactly what angle the patient was at. Here I'm really missing it.”* |
|  | Financial consideration is a barrier to use SMS | *“we don't have the ability or freedom generally to spend half an hour or an hour with people. Especially if you own your own business. If you're only making 100 bucks and hour and you have to pay the secretary and pay bills and those overheads…”* |
|  | The internship course requirements are a barrier to my use of SMS | *“Yeah. Exactly. We're assessed on how to adjust. We're assessed on how to put a modality in place. There's no active care component.”* |
|  | Patient characteristics that **restrict** the use of SMS: fear avoidance behavior, obstacles that patients face can vary, patient adherence to SMS, Patient motivation to use SMS, patient preferences | *“Patients that tend to have that fear avoidance and have that passive coping tend to be a lot tougher and have poor prognosis from a SMS strategy.”* |
|  | Educational Material contributes to patient adherence to SMS | *“it's of value to have something to give them that we've already discussed. It'll be a little more insightful and go into a little more detail than if you just give them a whole bunch of material that's going to sit on the to-read list.”* |
| Social Influence | The views of my clinician influence my use of SMS: restrict, facilitate, I consult my supervisory clinician on my use of SM and colleagues on my use of SMS/ Clinician preference affects student utilization of SMS | *“As I mentioned before we were pressured by faculty to prioritize spinal manipulation as our primary mode of treatment. That's a huge source of*  *bias in my opinion. And scientifically it's not literate at all.”* |
|  | Patient attitude (preferring passive care or highly motivated) restrict the use of SMS**/** patient acceptance and readiness for SMS affect my SMS practice | *“You’re so used to trying to get people to do something that saying, no don’t just stop… “* |
|  | SMS should be taught by people who actually believe in it | *“The important piece is it has to be included by people that actually believe in it, not by people that are in a union that necessitates them being there. Being taught rehab by someone who's not really in the rehab profession is pretty disappointing for someone like myself who is quite keen on something like that. And likewise I'm sure being taught pediatrics by people who are interested in working with kids by someone who isn't really involved in that aspect is disappointing.”* |
|  | Patient status/behavior makes me deliver SMS**/** Patient status/behavior restricts me from delivering SMS | *“I think a big thing in incorporating active care is really having a negotiation with the patient and seeing where they are in terms of what they want to do and what they're willing to do. Because you can give all the advice that you want, that could be helpful for anyone, but if they're not ready to accept it, or even do it, then you're not getting anywhere and you're just wasting each other's time. So just having that negotiation with them is important.”* |
| Emotion | I feel disappointed because of clinicians behaviours: prioritizing one treatment over another, non-aware of the guideline, don’t use SMS/ I feel stressed because of the internship course requirements don’t include SMS assessment | *“No but if you're anxious about giving them something, you should probably trust your anxiety and say 'look I want to get there but that's maybe not today, let's start with something small'. I think that if you're giving people something that makes them anxious you should probably step back and say 'why am I anxious?”* |
|  | I feel excited/good/optimistic about using SMS/ I feel anxious/nervous when use SMS**/** I feel concerned/frustrated when patient don’t adhere to SMS | *“I think it makes me feel like I've conducted myself well”* |
|  | I feel optimistic/motivated when I see patients benefitting from SMS**/** I feel hopeful when patient improved because of SMS/ I feel hopeless when patient not improved or have psychological overlay | *“Optimistic, excited. It's fun.”* |
| Behavioral Regulation | I adapt SMS for each patient individually**/** I monitor changes in patient's condition and change SMS components according to patient achievement | *“I have the tendency to just pull it away completely and start over as opposed to modifying. It could be just being a student, lack of experience. I'm not sure. But I don't exactly see something like that happen and say 'push' but I've seen our clinicians who are significantly more comfortable than some of us, that they'll see some of that quivering or pain or whatever and say 'It's okay, I want you to still keep going'. I think it's just experience”* |
|  | Providing feedback to patients on their progress between visits helps to motivate them**/** Providing feedback to patients on their progress during visits could be demotivating if they don't improve**/** providing feedback to patients on their progress between visits helps to motivate them**/** Assessing patients readiness for SMS is important to success it**/** patient motivation is needed | *“Because a lot of patients don't realize that they've had any progress until you show them. You know, like 'Can you see this?' and sometimes they're like 'Well no.' 'Let me show you think before picture.”* |
|  | SMS is a routine clinical practice | *“I try to incorporate that in every visit.”* |
|  | Using visual cues to reinforce patient increase the adherence to SMS**/** breaking down the SMS components facilitates SMS implementation**/** I will address the patient barrier to uptake SMS**/** we should assess patient SMS uptake/ designing a program to increase patient take of SMS | *“One thing I'd add to that is that if you have a clinic iPad for example. It's obviously confidential. Some of the patients I'll ask them 'Are you comfortable with me taking a picture of you on your phone?' So you can take these pictures home from your own visit. This is day 1, minute 1. We haven't done anything. This is what you look like. And I'll be honest, it's not very good. But by the end of that session, you started here. now you're here. We're not all the way where you need to go yet, but we're at least 50% better. That is very empowering to visually see.”* |
