# Supplementary material for: Promoting the use of self-management in novice chiropractors treating individuals with spine pain: the design of a theory-based knowledge translation intervention
Source: BMC Musculoskelet Disord. 2018 Sep 11;19:328. doi: 10.1186/s12891-018-2241-1 (PMC6134709; doi:10.1186/s12891-018-2241-1)
Supplement: Supplementary file 8 — “Mapping behaviour change techniques on key domains, proposed KT interventions and actions”. It provides a list of self-management-TDF barriers and the proposed KT interventions. (DOCX 19 kb) [file 12891_2018_2241_MOESM8_ESM.docx]

**Additional file 8:** Mapping behaviour change techniques on key domains, proposed KT interventions and actions

| Self-management-TDF barriers (BCTs) | KT Intervention | Actions |
| --- | --- | --- |
| Skills   - Lack of skills, new skills needed to acquire to deliver self-management   *Behavioral change techniques:*  *(Self-monitoring; Monitoring; Graded tasks (start easy); Increasing skills (problem solving; decision making, goal setting); Rehearsal of relevant skills; Modeling, demonstration of behavior by others; Homework; Perform behavior in other settings)*  Knowledge   - Clinicians and interns needs to acquire more knowledge on using SMS   *Behavioral change techniques:*  *Information regarding behaviour, outcome.* | - Perform behavior in different settings - Modeling, demonstration of behavior by others - Rehearsal - Opinion leaders (coaching) | - Context: More indebt training in self-management support (SMS) is needed for supervisory clinicians and interns - Aim: to improve on chiropractors’ and interns’ knowledge and skills in communicating with patients and using Brief Action Planning model (BAP) to deliver SMS - Strategy: provide structured training on the BAP - Opportunities: - Continuing education: offer clinicians/interns to register to courses on pain management that emphasis SMS to treat patients (Prompt: Chronic Pain Management (online learning) / 12-Credits / $5,000) - Live (and recorded) webinar and online educational module to provide information on SMS and demonstrate the delivery of the BAP to patients - Workshop: **educational** and **training** workshop led by certified self-management expert to coach the clinicians/interns in a small group on communication skills, SMS, and BAP principles. The clinicians/ interns will practice (rehearsal) the delivery of BAP approach during the workshop and they will get feedback on their practice - Distribute educational/instructional materials to clinicians/interns on how to deliver SMS including the BAP - Opinion Leader: identifying and training an opinion leader in each clinic to advice colleague clinicians and interns and facilitate the delivery of SMS (**two** supervisory clinicians at each clinic and **one** intern per MPT) - Educational outreach visits: send a certified/trained person (one of the research member) to the clinic to coach, monitor, and provide feedback to chiropractors and interns in person - Access an SM expert via Skype or phone number - Social media to provide information about SMS and the BAP - Record the session with patients to self-monitor SMS practice (to improve the skills and avoid the mistakes) |
| Social Influence   - Supervisory clinicians and patient influence clinicians/interns self-management practice   *Behavioral change techniques:*  *(Social processes of encouragement, pressure, and support; Social Support; Modeling/ Demonstration of behavior by others)* | - Opinion leaders | - Identify, train and certify an opinion leader in each clinic to facilitate the use of SMS (**two** supervisory clinicians at each clinic and **one** intern per MPT) - Encourage patients to use SMS by providing them the benefits of SMS |
|  | - Demonstration of behavior by expert clinician/opinion leader | - Workshop: educational and training workshop (interactive workshop) led by certified self-management person to coach clinicians and interns in a small group on communication skills, SMS, and BAP principles. Clinicians will practice (rehearsal) BAP during the workshop |
|  | - Persuasive Communication and Information regarding behavior outcome | - Encouraging collaboration between researchers and clinicians, and between clinicians and interns to discuss how best to administer SMS and related potential benefits |
|  | - Media mass for clinicians and patient | - Social media to provide information about the benefits of using SMS for both the clinicians/interns and their patients |
| Environmental Context and Resources   - Lack of time to deliver self-management for patients   *Behavioral change techniques:*  *(Environmental Changes; Time management)* | - Standardized electronic or printed materials as tools for facilitating the delivery of SMS | - Context: Lack of time among clinicians/interns is a barrier to the delivery of SMS to patients - Aim: developing a tool to facilitate and maximize the delivery of SMS in clinical settings - Opportunity: - Provide clinicians with material (algorithm, poster) to ease understanding, memory and use of the BAP. The algorithm will be provided to participants in paper format. The poster will be in places where clinicians/interns meet with patients. The material will be presented in the training workshop to familiarize clinicians with the information. - Providing the clinicians with a website or material summarizing the SMS components (exercises, diet program, …) |
| Emotion   - Feeling of anxiety/ concerned/nervous when use SMS   *Behavioral change techniques:*  *(Stress Management, Social support (emotional); Coping strategies)* | - Opinion leaders - Perform behavior in different settings - Modeling, demonstration of behavior by others | - Context: Clinicians and interns stated that they have some anxiety and concern regarding the use of SMS with certain patients - Aim: minimize the anxiety among clinicians/interns by maximizing their capabilities of using SMS - Opportunity: - Identify, train and certify an opinion leader in self-management in each clinic to support clinicians/interns in using SMS and to uncover challenges related to using of SMS and find potential solutions to address these barriers (**two** supervisory clinicians at each clinic and **one** intern per MPT) - Continuing education: offer clinicians/interns to register to courses on pain management that emphasis self-management to treat patients (Prompt: Chronic Pain Management (online learning) / 12-Credits / $5,000) - Workshop: educational and **training** workshop led by certified self-management expert to coach clinicians/interns in a small group on SMS, communication skills, motivational interviewing, and BAP principles. The clinicians/ interns will practice (rehearsal) the delivery of BAP approach during the workshop |
| Memory, attention & decision making   - Few clinicians/ interns don’t follow guidelines to use SMS   *Behavioural change techniques:*  *planning/implementation; Prompts/Triggers/Cues; Motivational Interviewing* | - Opinion Leaders - Persuasive Communication and Information regarding behavior outcome - Information Provision - Instructions | - Context: Few clinicians/interns do not follow guideline recommendations on the use of SMS - Aim: to support clinicians/interns to use evidence and SMS to inform clinical decision - Opportunities: - Continuing education: offer clinicians/interns to register to courses on pain management that emphasis SMS to treat patients (Prompt: Chronic Pain Management (online learning) / 12-Credits / $5,000) - Live (and recorded) webinar and online educational module to provide information SMS and demonstrate the delivery of the BAP to patients - Workshop: educational and **training** workshop led by certified self-management expert to coach the clinicians/interns in a small group on BAP self-management - Distribute educational/instructional materials to clinicians/interns on how to deliver SMS guiding by BAP - Opinion Leader: identify and train an opinion leader in each clinic to advice chiropractors and interns, and facilitate the delivery of SMS (one on one coaching) (**two** supervisory clinicians at each clinic and **one** intern per MPT) - Educational outreach visits: send a certified/trained person (one of the research member) to the clinic to coach, monitor, and provide feedback to chiropractors and interns in person - Access an SM expert via Skype or phone number - Social media to provide information about the benefits of using SMS for both the clinicians/interns and their patients |
| Behavioural Regulation   - SMS needs to be adapted for each patients individually   *Behavioural change techniques:*  *planning/implementation; Prompts/Triggers/Cues* | - **Action Planning:**   BAP algorithm | - Context: Clinicians and interns individualize SMS - Aim: to facilitate individualize the SMS for patients - Opportunities: - Live (and recorded) webinar and online educational module to provide information SMS and demonstrate the delivery of the BAP to patients - Workshop: educational and **training** workshop led by certified self-management expert to coach the clinicians/interns in a small group on self-management using BAP. The clinicians/ interns will practice the delivery of BAP approach during the workshop - Distribute educational/instructional materials to clinicians/interns on how to deliver SMS guiding by BAP - Opinion Leader: identify and train an opinion leader in each clinic to advice chiropractors and interns, and facilitate the delivery of SMS (**two** supervisory clinicians at each clinic and **one** intern per MPT) - Educational outreach visits: send a certified/trained person (one of the research member) to the clinic to coach, monitor, and provide feedback to chiropractors and interns in person - Access an SM expert via Skype or phone number - Social media to provide information about the benefits of using SMS for both the clinicians/interns and their patients |
