# Supplementary material for: Promoting the use of self-management in novice chiropractors treating individuals with spine pain: the design of a theory-based knowledge translation intervention
Source: BMC Musculoskelet Disord. 2018 Sep 11;19:328. doi: 10.1186/s12891-018-2241-1 (PMC6134709; doi:10.1186/s12891-018-2241-1)
Supplement: Supplementary file 9 — “Final Selection of KT Intervention Components and Related Learning”. It provides the final selection of KT intervention for clinicians and interns to promote the use of self-management support in the clinic. (DOCX 14 kb) [file 12891_2018_2241_MOESM9_ESM.docx]

**Additional file 9: Final selection of KT intervention components and related learning objectives**

1. Providing chiropractors and interns with supportive handouts summarizing how to use the SMS guiding by BAP.

- Paper-based (BAP flow chart and guide)

1. Webinar (55 minutes):

- Learn the process and the attitudes of self-management strategies
- Become familiar with the process of Brief Action Planning
- Become familiar with the attitudes to adopt when using it with a patient

1. Online educational module on the BAP: (22-minute learning module with segmented video to help apply material learned on self-management and Brief Action Planning).

- Become familiar with the process of Brief Action Planning
- Become familiar with the attitudes to adopt when using it with a patient. BAP motivational interviewing led by interns/clinicians

1. Clinical vignettes: (different BAP scenarios):

- Become familiar with using the Brief Action Planning
- Become familiar with the attitudes to adopt when using it with a patient.

1. Training workshop (one-full day)

- Have more opportunity to practice SMS
- Get personalized feedback

1. Opinion leader

- Advise colleagues about SMS practice
- Ease the delivery of SMS
